# Neuropathic-like knee pain and associated risk factors: a cross-sectional study in a UK community sample

**DOI:** 10.1186/s13075-018-1717-6

**Published:** 2018-09-27

**Authors:** Gwen Sascha Fernandes, Ana Marie Valdes, David Andrew Walsh, Weiya Zhang, Michael Doherty

**Affiliations:** 1 Academic Rheumatology, Division of Rheumatology, Orthopedics and Dermatology, Nottingham City Hospital, University of Nottingham, Clinical Sciences Building, Nottingham, NG5 1PB UK; 2 Arthritis Research UK Centre for Sports, Exercise and Osteoarthritis, Queen’s Medical Centre, Derby Road, Nottingham, NG7 2UH UK; 3 Arthritis Research UK Pain Centre, University of Nottingham, Nottingham, NG5 1PB UK; 4 NIHR Nottingham Biomedical Research Centre, University of Nottingham, Nottingham, NG5 1PB UK

**Keywords:** Neuropathic pain, Osteoarthritis, Risk factors, Pain catastrophising, Anxiety, Depression

## Abstract

**Background:**

Neuropathic-like knee pain (NKP) is often reported in individuals with knee pain (KP), but the contribution of specific central and peripheral risk factors to NKP has not been studied previously. The aims of the present study were to determine the prevalence of NKP in a community-derived sample with KP and to identify risk factors associated with NKP.

**Methods:**

A cross-sectional study was undertaken (*n* = 9506) in the East Midlands community among responders (aged 40+ years) to a postal questionnaire. Questions included KP severity (numerical rating scale) and type (neuropathic versus nociceptive) using the modified painDETECT questionnaire, as well as age, body mass index (BMI), significant knee injury, widespread pain, pain catastrophising and fatigue. Multinomial regression analysis was used to determine ORs and 95% CIs. Risk factors were categorised into central and peripheral, and proportional risk contribution (PRC) and 95% CI were estimated using ROC.

**Results:**

KP was reported in 28.2% of responders, of whom 13.65% had NKP (i.e., 3.9% of the total population). Women reported more NKP. After adjustment for age, gender, BMI and pain severity, definite NKP showed associations (aOR, 95% CI) with fibromyalgia (4.07, 2.49–6.66), widespread pain (1.93, 1.46–2.53), nodal osteoarthritis (1.80, 1.28–2.53), injury (1.50, 1.12–2.00), pain catastrophising (5.37, 2.93–9.84) and fatigue (5.37, 3.08–9.35) compared with non-NKP participants. Although only central risk factors contributed to NKP (PRC 8%, 95% CI 2.5–12.5 for central vs. PRC 3%, 95% CI −0.25 to 7.5 for peripheral), both central and peripheral risk factors contributed equally to non-NKP (PRC 10%, 95% CI 5–20 for both).

**Conclusions:**

NKP appears to be driven largely by central risk factors and may require different prevention/treatment strategies.

**Trial registration:**

ClinicalTrials.gov, NCT02098070. Registered on 27 March 2014.

## Background

Knee pain (KP) is a major cause of disability worldwide. In the United Kingdom (UK) approximately 1 in 4 people aged 55 years and over report prevalent KP [1]. KP is often attributed to localised tissue insult that causes nociceptive pain. However, recent data suggest that localised damage to the nervous system and nerve fibres around a joint could result in neuropathic-like knee pain (NKP) that modifies the KP experience [2]. KP can also be modified by central sensitisation following chronic nociceptor stimulation and alterations in central pain transmitting neurons and can result in NKP characteristics [3]. This could be induced by localised insult to the joint, by comorbid conditions or by psychological factors that modify pain physiology and descending pain modulation. However, such modifying factors are rarely studied and are often omitted in clinical assessments of KP [4].

KP can be broadly categorised into nociceptive (inflammatory or mechanical local mechanisms); neuropathic, involving nerve damage (and potentially involving central mechanisms); and idiopathic pain with no identified cause (presumably driven predominantly by central factors) [5]. Being able to correctly identify different types of KP could have implications for treatment selection and management pathways. KP is the main complaint of knee osteoarthritis (KOA) [6] and is the primary reason patients seek treatment. As Hadler eloquently wrote, ‘Knee pain is the malady, not osteoarthritis’ [7]. Furthermore, half of those who complain of KP have no definite radiographic evidence of KOA [8], and 20% of people with KOA have persistent severe KP even after total knee replacement [9]. These findings suggest that central factors other than severity of local structural KOA influence KP experience and that identifying and addressing these central factors associated with NKP features, particularly early in the disease, could benefit people with such KP.

NKP associates with characteristic symptoms and pain qualities, including burning pain, tingling or prickling, mechanical and thermal hyperalgesia, allodynia, paroxysmal pain and numbness. Although quantitative sensory testing (QST) is helpful in confirming abnormal pain thresholds, this is impractical for widespread use within a community setting [10]. There are a number of screening tools and questionnaires, such as the SLANNS, painDETECT questionnaire (PDQ) and DN4 (Douleur Neuropathique 4), that use descriptions of pain location, intensity, frequency and pain quality to determine whether pain is likely to be neuropathic or nociceptive [11, 12]. The PDQ was subsequently modified (mPDQ) for use in specific areas of the body, such as the knee, with good face and content validity and good correlation with QST signs of central sensitisation [13, 14]. Hochman et al. [10] reported that one-third of participants with KOA (*n* = 259) described their pain using neuropathic descriptors and were more likely to be younger and female, with higher pain and osteoarthritis (OA) severity as well as longer OA duration, than those who did not use such descriptors. The research suggests the existence of subgroups of participants with shared characteristics that exhibit NKP symptoms. Should that be the case, targeting treatment to the underlying pain mechanism could have the potential to improve pain management and to improve quality of life with individualised treatment interventions. The objectives of this study were to (1) determine the prevalence of NKP in community-derived people with KP and (2) identify risk factors specifically associated with NKP.

## Methods

### Study design

The Nottingham Knee Pain and Health in the Community (KPIC) study is an ongoing prospective cohort study in the East Midlands region of the UK [15]. The current study used baseline KPIC data to identify people with KP and the proportion of those who reported NKP. A case-control study was conducted with three groups: NKP, non-NKP and no KP. The study was approved by the Nottingham Research Ethics Committee 1 (NREC reference 14/EM/0015) and registered with ClinicalTrials.gov (NCT02098070).

### Sample size

#### Source population

The survey comprised a community-derived sample regardless of whether subjects had experienced KP. A postal questionnaire was developed on the basis of a review of items in previously published questionnaires [16, 17]. Further details on questionnaire logistics and sample size calculations have been published previously [15].

#### NKP prevalence and risk factors

According to a NKP prevalence in people with KP of 28% (±8%) based on a Canadian community population sample [10], a minimum of 85 participants with KP were required to yield a power of 90% with a 0.05 significance level.

### Participants

#### Inclusion criteria

The inclusion criteria were all men and women aged 40 years and over, located on their general practitioner (GP) register, regardless of KP status.

#### Exclusion criteria

Exclusion criteria were inability to give informed consent and presence of a terminal illness or severe mental illness. Eligibility was decided by the GPs in each practice. The questionnaire was accompanied by a covering letter from the GP introducing the study and its objectives. Return of a completed questionnaire in a pre-paid envelope to Academic Rheumatology (City Hospital, Nottingham) was taken as implicit consent.

### Questionnaire survey

The questionnaire was constructed to capture detailed information about the participants, as well as their medical history and risk factors for KOA [18]. A validated screening question was used to determine presence of current KP: ‘Have you ever had knee pain for most days of the past one month?’ [19, 20]. Additionally, a body pain manikin [21] was used to locate and define pain in other body regions, allowing definition of widespread pain based on criteria proposed by the American College of Rheumatology which require presence of pain in all four quadrants as well as the axial skeleton [22]. The mPDQ was chosen to identify NKP (PDQ scores ≥ 13 as possible NKP and ≥ 19 as definite NKP) [13]. On the basis of face validity of the questionnaire content, previous literature [23] and consensus among the authors, the data on risk factors obtained from the questionnaire were divided into three groups: peripheral (related to structural changes in and around the knee joint, such as significant injury and nodal OA [which associates with likelihood of structural KOA]), central (related to pain experience and physiology such as anxiety, depression, fatigue, Pain Catastrophising Scale [PCS] and self-reported GP-diagnosed fibromyalgia) and comorbidities (such as hyperlipidaemia, diabetes and widespread pain as defined using the body pain manikin [15]). Further details on each exposure measured included KP severity, constitutional knee alignment, nodal OA, 2D:4D (index:ring) digit ratio, anxiety, depression, fatigue and pain catastrophising, and high-risk occupations can be found in the published study protocol [15]. Exposures such as 2D:4D digit ratio and nodal OA are recognised risk factors for knee pain and knee OA [1, 13, 15] and were included in order to identify as many possible associations and confounders as possible [10, 13, 14].

### Statistical analyses

Categorical variables were reported as frequencies and continuous variables as mean and SD. OR and 95% CI were calculated using multinomial logistic regression for three-group comparisons (NKP, non-NKP and no KP). Each risk factor was adjusted for the same confounding factors (age, body mass index [BMI], gender and pain severity). Statistical significance was defined as *p* < 0.05. There were very few missing data at random (e.g., where BMI was not reported by a participant), so imputation or modelling was not undertaken for occasional missing values. All variables were reported as dichotomous data, with the exception of PCS and fatigue scores. PCS was reported as per official cut-offs in tertiles (< 18 [lowest tertile], 18–24 [middle tertile], ≥24 [highest tertile]). Fatigue data were reported using a Likert-style scale and were categorised as lowest tertile (never), middle tertile (seldom and sometimes) and highest tertile (often and always).

We also used ROC to calculate AUC, from which proportional risk contribution (PRC) of central risk factors, peripheral risk factors and comorbidities was derived. ROC curves were based on the multivariate logistic regression model with definite NKP as an outcome compared with non-NKP. Firstly, we built the full risk model for definite NKP with an ROC curve (ROC1). Secondly, we removed the exposure(s) of interest to examine the contribution of the exposure(s) through the reduction of the ROC curve (i.e., the partial ROC [ROC2]). Thirdly, we calculated the PRC using the following formula:

$$ \mathrm{PRC}=\left[\mathrm{ROC}1-\mathrm{ROC}2\right]/\left[\mathrm{ROC}1-0.5\right] $$

The 95% CI of PRC was calculated according to 95% CIs of ROC1 and ROC2. We also calculated ROC curves based on non-NKP as an outcome compared with no KP to determine the PRC of peripheral and central risk factors and comorbidities. PRCs for each of the groups (central and peripheral risk factors and comorbidities) indicate the contribution of each to the outcome of definite NKP. The ROC curves were generated using STATA software (StataCorp, College Station, TX, USA) with the *roctab* command and combined graphically [24]. All analysis was conducted using Stata IC version 14 on the Windows 7 operating system, and power calculations were undertaken using Power and Precision version 2.1 software (Biostat, Englewood, NJ, USA).

## Results

Of the 40,505 mailed questionnaires, 9506 (23.4%) completed questionnaires were returned which met the inclusion criteria. The characteristics of these participants were compared with those from other national UK cohorts (Appendix). The data showed that the KPIC population is representative of the UK general population in terms of age, percentage of women and BMI (Appendix).

**Fig. 1:**
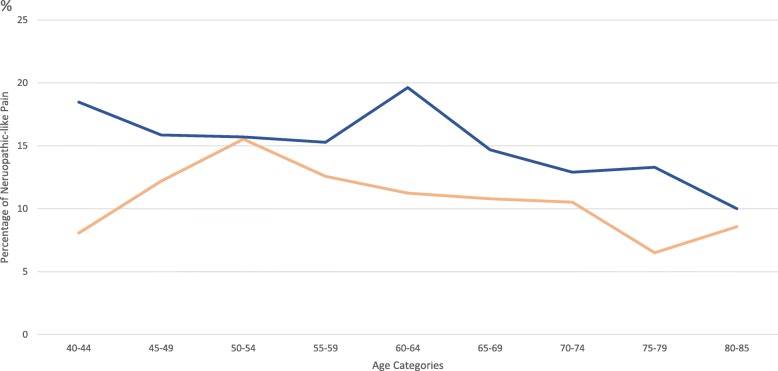
The prevalence of definite neuropathic-like knee pain in the knee pain population in Nottingham Knee Pain and Health in the Community study, by gender and in age categories

Of the responders, 2681 participants (28.3%) reported KP for most days of the past month. Of these KP participants, 366 (13.65% of those with KP, 3.9% of the total KPIC) had NKP. Proportions of 16.6% of women with KP and 11.6% of men with KP reported definite NKP. Women also reported more NKP in almost every age category than men and peaked 10 years later in the age range of 60–64 years than men in the age range of 50–54 years (Fig. 1).

Comparison of the participants in the four categories (definite NKP, possible NKP, non-NKP and no KP) showed that those with definite NKP were more likely to be younger, female and have greater BMI (*p* < 0.01). They were also more likely to self-report a diagnosis of fibromyalgia, hypertension, hyperlipidaemia and diabetes; report significant knee injury; work in high-risk occupations; present with nodal OA and widespread pain; and have higher scores for anxiety and depression than people without KP (*p* < 0.001). These results are presented in Table 1.

**Table 1:** Comparison of all characteristics of four groups within the Nottingham Knee Pain and Health in the Community study community

Characteristics	No KP (*n* = 6822)	Non-NKP (*n* = 1685)	Possible NKP (*n* = 462)	Definite NKP (*n* = 366)	*P* value
Age, yr, mean (±SD)	62.12 (10.64)	61.94 (10.41)	61.80 (10.32)	61.13 (9.96)	0.03*
Body mass index, kg/m^2^, mean (±SD)	26.61 (4.76)	28.30 (5.42)	29.84 (6.39)	31.49 (7.77)	< 0.001*
Female gender, *n* (%)	3806 (55.80.09)	956 (56.74)	258 (55.84)	242 (66.12)	0.02*
High-risk occupation, *n* (%)	2660 (38.99)	783 (46.47)	271 (58.66)	193 (52.73))	< 0.001*
Fibromyalgia, *n* (%)	101 (1.48)	52 (3.09)	26 (5.63)	57 (15.57)	< 0.001*
Hypertension, *n* (%)	1890 (27.70)	555 (32.94)	183 (39.61)	146 (39.89)	< 0.001*
High cholesterol, *n* (%)	1773 (25.99)	495 (29.38)	156 (33.77)	143 (39.07))	< 0.001*
Stroke, *n* (%)	174 (2.55)	51 (3.02)	14 (3.03)	15 (4.10)	0.05
Diabetes, *n* (%)	550 (8.06)	172 (10.21)	72 (15.58)	73 (19.95))	< 0.001*
2D:4D digit ratio, *n* (%)	3256 (47.73)	792 (47.00)	232 (50.22)	142 (38.80)	0.03*
Nodal OA, *n* (%)	632 (9.62)	234 (14.60)	79 (18.20)	88 (25.36)	< 0.001*
Significant injury, *n* (%)	972 (14.25)	478 (28.38)	165 (35.71)	130 (35.52)	< 0.001*
Widespread pain, *n* (%)	1072 (15.71)	537 (31.87)	209 (45.24)	198 (54.10)	< 0.001*
Anxiety, *n* (%)	754 (11.05)	298 (17.70)	148 (32.03)	191 (52.19)	< 0.001*
Depression, *n* (%)	266 (3.90)	142 (8.43)	91 (19.70)	133 (36.34))	< 0.001*

*Abbreviations: KP* Knee pain, *NKP* Neuropathic-like knee pain, *OA* Osteoarthritis

* is statistical significance <0.05

We compared definite NKP participants with no-KP participants to determine which factors were associated with definite neuropathic-like symptoms compared with no KP. Unadjusted and adjusted ORs (for age, BMI, gender and pain severity) for definite NKP compared with no-KP participants were calculated for each risk factor (Table 2). The results of the multinomial regression analysis comparing NKP with no KP showed significant associations, particularly for central factors such as fibromyalgia (aOR 3.19, 95% CI 1.70–5.98), anxiety (aOR 2.48, 95% CI 1.76–3.48), depression (aOR 2.62, 95% CI 1.70–4.04), highest tertile of pain catastrophising (aOR 5.00, 95% CI 2.68–9.36) and highest tertile of fatigue (aOR 5.11, 95% CI 2.86–9.15) (Table 2). The most significant result for peripheral risk factors was significant knee injury and the presence of nodal OA (1.44 [1.04–1.99] and 1.76 [1.19–2.63], respectively).

**Table 2:** Regression results of definite neuropathic-like knee pain versus possible neuropathic-like knee pain, non-neuropathic-like knee pain and no knee pain in the whole Nottingham Knee Pain and Health in the Community study population

		OR (95% CI)					
	No KP	Non-NKP		Possible NKP		Definite NKP	
		Crude	Adjusted	Crude	Adjusted	Crude	Adjusted
	(*n* = 6822)	(*n* = 1685)		(*n* = 462)		(*n* = 366)	
Age	1 (referent)	0.99 (0.99; 1.00)	–	0.99 (0.99;1.00)	–	0.99 (0.98;1.00)	–
Body mass index	1	**1.06 (1.06; 1.08)**	**–**	1.11 (1.09;1.13)	**–**	1.15 (1.13;1.17)	**–**
Gender	1	1.04 (0.93; 1.16)	–	1.03 (0.85; 1.24)	–	1.57 (1.25; 1.96)	–
Pain severity	1	**1.70 (1.64; 1.77)**	–	2.28 (2.15; 2.53)	–	3.44 (3.16; 3.74)	–
Peripheral factors							
High-risk occupation	1	**1.36 (1.22; 1.51)**	0.88 (0.74; 1.05)	2.22 (1.83; 2.69)	**1.38 (1.06; 1.80)**	1.75 (1.41; 2.16)	1.04 (0.77; 1.42)
Significant injury	1	**2.40 (2.12; 2.73)**	0.93 (0.78; 1.12)	3.41 (2.78; 4.18)	1.29 (0.98; 1.69)	3.53 (2.81; 4.44)	**1.44 (1.04; 1.99)**
Nodal OA	1	**1.60 (1.36; 1.89)**	1.01 (0.78; 1.30)	2.09 (1.62; 2.70)	1.34 (0.93; 1.93)	3.19 (2.47; 4.12)	**1.76 (1.19; 2.63)**
2D:4D digit ratio	1	0.99 (0.99; 1.00)	0.99 (0.99; 1.00)	0.99 (0.99; 1.00)	0.99 (0.99; 0.99)	1.00 (0.99; 1.00)	0.99 (0.99; 1.00)
Central factors							
Anxiety	1	**1.73 (1.49; 2.00)**	0.8 (0.63; 1.00)	3.79 (3.08; 4.68)	**1.42 (1.04; 1.94)**	8.78 (7.06; 10.93)	**2.48 (1.76; 3.48)**
Depression	1	**2.27 (1.84; 2.80)**	0.84 (0.60; 1.19)	6.04 (4.66; 7.84)	**1.71 (1.13; 2.59)**	14.06 (11.00; 17.99)	**2.62 (1.70; 4.04)**
Pain Catastrophising Scale							
Lowest tertile	1	–	–				
Middle tertile		**2.00 (1.75; 2.23)**	1.19 (0.97; 1.47)	4.93 (3.35; 7.24)	**2.45 (1.57; 3.82)**	4.04 (2.14; 7.62)	1.60 (0.80; 3.23)
Highest tertile		**2.53 (2.20; 2.91)**	0.87 (0.69; 1.09)	14.98 (10.44; 21.49)	**3.33 (2.16; 5.14)**	40.84 (23.36; 71.42)	**5.00 (2.68; 9.36)**
Fatigue							
Lowest tertile	1	**–**	**–**				
Middle tertile		**1.94 (1.70; 2.22)**	1.01 (0.83; 1.24)	2.75 (2.04; 3.69)	1.14 (0.79; 1.65)	6.26 (3.72; 10.55)	**2.38 (1.31; 4.33)**
Highest tertile		**2.80 (2.44; 3.23)**	0.97 (0.77; 1.22)	7.77 (5.86; 10.30)	**1.86 (1.29; 2.68)**	31.20 (19.02; 51.21)	**5.11 (2.86; 9.15)**
Fibromyalgia	1	**2.11 (1.51; 2.97)**	0.79 (0.47; 1.32)	3.97 (2.55; 6.17)	1.27 (0.66; 2.42)	12.28 (8.70; 17.32)	**3.19 (1.70; 5.98)**
Comorbidities							
Hypertension	1	**1.28 (1.14; 1.44)**	1.00 (0.82; 1.21)	1.71 (1.41; 2.07)	1.18 (0.88; 1.57)	1.73 (1.40; 2.15)	1.09 (0.78; 1.53)
Hyperlipidaemia	1	**1.18 (1.05; 1.33)**	1.05 (0.86; 1.28)	1.45 (1.19; 1.77)	1.17 (0.87; 1.56)	1.83 (1.47; 2.27)	**1.44 (1.03; 2.01)**
Stroke	1	1.19 (0.87; 1.64)	1.12 (0.66; 1.89)	1.19 (0.69; 2.07)	1.18 (0.55; 2.50)	1.63 (0.95; 2.80)	1.13 (0.48; 2.68)
Diabetes	1	**1.30 (1.08; 1.55)**	0.91 (0.67; 1.22)	2.10 (1.61; 2.75)	1.20 (0.80; 1.80)	2.84 (2.17; 3.72)	1.38 (0.88; 2.15)
Multiple regional pain	1	**2.50 (2.22;2.83)**	0.97 (0.81; 1.18)	4.43 (3.65; 5.38)	**1.60 (1.23; 2.09)**	6.32 (5.10; 7.84)	**1.90 (1.40; 2.60)**
Pain experience							
ICOAP overall	1	**1.11 (1.10; 1.12)**	**1.08 (1.07; 1.10)**	1.15 (1.14; 1.16)	**1.11 (1.10; 1.13)**	1.19 (1.18; 1.20)	**1.14 (1.13; 1.16)**
ICOAP intermittent	1	**1.09 (1.08; 1.10)**	**1.06 (1.05; 1.07)**	1.13 (1.12; 1.14)	**1.08 (1.07; 1.09)**	1.17 (1.15; 1.18)	**1.10 (1.09; 1.12)**
ICOAP constant	1	**1.10 (1.09; 1.10)**	**1.07 (1.06; 1.08)**	1.13 (1.13; 1.14)	**1.10 (1.09; 1.11)**	1.18 (1.16; 1.19)	**1.12 (1.11; 1.14)**

*Abbreviations: ICOAP* Intermittent and Constant Osteoarthritis Pain measure, *KP* Knee pain, *NKP* Neuropathic-like knee pain, *OA* Osteoarthritis

No KP is no knee pain; non-NKP is non-neuropathic-like knee pain; likely NKP is likely neuropathic pain; and definite NKP is definite neuropathic-like knee pain

The no KP group is the referent group, and hence a ‘1’ represents this in the table

*Note:* Significant associations are highlighted in bold. For comparison purposes, we only present crude and age-, gender-, body mass index- and pain severity-adjusted OR for each factor

We compared definite NKP participants with non-NKP participants to determine which factors were associated with definite neuropathic-like symptoms compared with non-neuropathic-like knee pain. Unadjusted and adjusted ORs for definite NKP versus non-NKP were also calculated for each risk factor (Table 3). After adjustment for age, BMI, gender and KP severity, definite NKP was significantly associated with two peripheral risk factors (injuries [aOR 1.50, 95% CI 1.12–2.00] and nodal OA [aOR 1.80, 95% CI 1.28–2.53]) and all measured central risk factors (fibromyalgia [aOR 4.07, 95% CI 2.49–6.66], anxiety [aOR 3.17, 95% CI 2.38–4.23], depression [aOR 2.99, 95% CI 2.14–4.19], pain catastrophising [aOR 5.37, 95% CI 2.93–9.84] and fatigue [aOR 5.37, 95% CI 3.08–9.35]). There were also associations with markers of metabolic syndrome, such as diabetes (aOR 1.52, 95% CI 1.04–2.23) and hyperlipidaemia (aOR 1.36, 95% CI 1.01–1.84).

**Table 3:** Regression results of definite neuropathic-like knee pain versus possible- and non-neuropathic-like knee pain

		OR (95% CI)			
	Non-NKP	Possible NKP		Definite NKP	
		Crude	Adjusted	Crude	Adjusted
	(*n* = 1685)	(*n* = 462)		(*n* = 366)	
Age	1 (Reference)	0.99 (0.99; 1.00)	–	0.99 (0.98; 1.00)	–
Body mass index	1	1.04 (1.02; 1.06)	**–**	1.08 (1.06; 1.10)	–
Gender	1	0.98 (0.79; 1.21)	–	1.50 (1.18; 1.91)	–
Pain severity	1	1.37 (1.31–1.45)	–	2.08 (1.93; 2.25)	–
Putative factors					
High-risk occupation	1	1.63 (1.33; 2.01)	**1.57 (1.24; 1.99)**	1.29 (1.03–1.61)	1.17 (0.89; 1.54)
Significant injury	1	1.42 (1.14; 1.77)	**1.36 (1.07; 1.73)**	1.47 (1.15; 1.88)	**1.50 (1.12; 2.00)**
2D:4D digit ratio	1	1.00 (0.99; 1.00)	0.99 (0.99; 1.00)	1.00 (0.99; 1.00)	0.99 (0.99; 1.00)
Nodal OA	1	1.30 (0.98; 1.72)	1.34 (0.98; 1.83)	1.99 (1.50; 2.63)	**1.80 (1.28; 2.53)**
Comorbidities					
Hypertension	1	1.34 (1.08; 1.65)	1.18 (0.92; 1.52)	1.35 (1.07; 1.70)	1.09 (0.80; 1.47)
Hyperlipidaemia	1	1.22 (0.98; 1.53)	1.12 (0.86; 1.44)	1.54 (1.22; 1.95)	**1.36 (1.01; 1.84)**
Stroke	1	1.00 (0.55; 1.83)	0.97 (0.52; 1.84)	1.37 (0.76; 2.46)	0.92 (0.44; 1.92)
Diabetes	1	1.62 (1.20; 2.19)	1.32 (0.94; 1.85)	2.19 (1.62; 2.96)	**1.52 (1.04; 2.23)**
Multiple regional pain	1	1.76 (1.43; 2.18)	**1.63 (1.29; 2.05)**	2.52 (2.00; 3.17)	**1.93 (1.46; 2.53)**
Central factors					
Anxiety	1	2.20 (1.74; 2.76)	**1.79 (1.38; 2.32)**	5.08 (3.99; 6.45)	**3.17 (2.38; 4.23)**
Depression	1	2.66 (1.99; 3.55)	**1.96 (1.42; 2.70)**	6.20 (4.71; 8.15)	**2.99 (2.14; 4.19)**
Pain Catastrophising					
Scale					
Lowest tertile	1	Reference			
Middle tertile		2.46 (1.64; 3.67)	**2.03 (1.32; 3.10)**	2.01 (1.05; 3.84)	1.31 (0.66; 2.60)
Highest tertile		5.91 (4.05; 8.62)	**3.63 (2.40; 5.48)**	16.12 (9.12; 28.49)	**5.37 (2.93; 9.84)**
Fatigue					
Lowest tertile	1	Reference			
Middle tertile		1.41 (1.03; 1.93)	1.15 (0.82; 1.62)	3.22 (1.89; 5.48)	**2.45 (1.38; 4.38)**
Highest tertile		2.77 (2.05; 3.75)	**1.91 (1.38; 2.66)**	11.12 (6.69; 18.47)	**5.37 (3.08; 9.35)**
Fibromyalgia	1	1.87 (1.16; 3.03)	1.56 (0.92; 2.66)	5.79 (3.90; 8.61)	**4.07 (2.49; 6.66)**
Treatment					
Opioids	1	1.98 (1.54; 2.54)	**1.46 (1.11; 1.92)**	3.03 (2.35;3.91)	**1.70 (1.26; 2.31)**
NSAIDs	1	1.22 (0.82; 1.81)	1.01 (0.66; 1.56)	2.03 (1.40; 2.95)	**1.75 (1.13; 2.72)**
OTC painkillers	1	1.66 (1.33; 2.07)	**1.39 (1.09; 1.78)**	1.76 (1.38; 2.24)	**1.28 (0.96; 1.71)**
Aspirin	1	1.31 (0.96; 1.79)	1.12 (0.79; 1.60)	1.66 (1.20; 2.28)	1.40 (0.94; 2.09)
Current statin use	1	0.99 (0.99; 1.00)	0.99 (0.99; 1.00)	0.99 (0.99; 1.00)	**1.00 (0.99; 1.00)**
Statin use ever	1	0.99 (0.99;0.99)	0.99 (0.99; 1.00)	0.99 (0.99; 0.99)	0.99 (0.99; 1.00)
Injection	1	2.04 (1.59; 2.61)	**1.66 (1.27; 2.18)**	3.52 (2.74; 4.52)	**2.39 (1.76; 3.23)**
Pain experience					
ICOAP overall	1	1.04 (1.03; 1.05)	**1.03 (1.02; 1.04)**	1.08 (1.07; 1.09)	**1.05 (1.04; 1.07)**
ICOAP intermittent	1	1.04 (1.03; 1.04)	**1.02 (1.02; 1.03)**	1.07 (1.06; 1.08)	**1.04 (1.04; 1.05)**
ICOAP constant	1	1.04 (1.03; 1.04)	**1.05 (1.04; 1.06)**	1.07 (1.06; 1.08)	**1.05 (1.04; 1.06)**

*Abbreviations: ICOAP* Intermittent and Constant Osteoarthritis Pain measure, *NKP* Neuropathic-like knee pain, *NSAIDs* Non-steroidal anti-inflammatory drugs, *OA* Osteoarthritis, *OTC* Over the counter

Non-NKP is non-neuropathic-like knee pain; likely NKP is likely neuropathic pain; and definite NKP is definite neuropathic-like knee pain

The non-KP group is the referent group, and hence a ‘1’ represents this in the table

*Note:* Significant associations are highlighted in bold. For comparison purposes, we only present crude and age-, gender-, BMI- and pain severity-adjusted OR for each factor

**Fig. 2:**
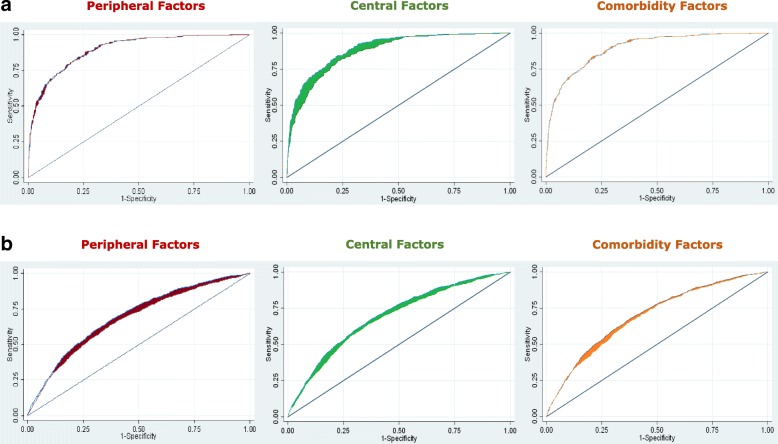
**a** Proportional risk contribution (PRC) of peripheral, central and comorbidity risk factors of definite neuropathic-like knee pain (NKP) vs. non-NKP. **b** PRC of peripheral, central and comorbidity risk factors of non-NKP vs. no knee pain (KP). *Blue* = full model. *Red* = PRC from peripheral factors. *Green* = PRC from central factors. *Orange* = PRC from comorbidities. *Note:* PRC was estimated from full ROC analysis. Full models included age, gender, body mass index and pain severity, nodal OA + significant knee injury (peripheral factors), anxiety + depression + Pain Catastrophising Scale, fibromyalgia and fatigue (central factors) and diabetes + hyperlipidaemia + widespread pain (comorbidities). Pain severity variable was removed from the second ROC analysis comparing non-NKP vs. no KP participants

The ROC for the full model for definite NKP compared with non-NKP, including central and peripheral risk factors and comorbidities was 0.90 (0.88; 0.92) with the PRC of peripheral, central and comorbidity risk factors as 3%, 8% and 3%, respectively. The ROC for the full model for non-NKP compared with no KP was 0.70 (0.69; 0.72) with the PRC of peripheral, central and comorbidity risk factors as 10%, 10% and 5%, respectively. These ROC graphs are presented in Fig. 2, with further details presented in Table 4.

**Table 4:** ROC and 95% CI for risk factor groups comparing different participants based on neuropathic-like knee pain profile

	ROC	95% CI	PRC (%)	PRC 95% CI
Definite NKP vs. non-NKP				
Full*	0.90	0.88; 0.92	100%	–
Without peripheral	0.89	0.87; 0.91	3%	−2.5; 7.5
Without central	0.87	0.85; 0.89	8%	2.5; 12.5
Without comorbidities	0.89	0.88; 0.91	3%	−2.5; 5
Non-NKP vs. no KP				
Full	0.70	0.69; 0.72	100%	–
Without peripheral	0.68	0.66; 0.69	10%	5; 20
Without central	0.68	0.66; 0.69	10%	5; 20
Without comorbidities	0.69	0.67; 0.70	5%	0; 15

*Abbreviations: KP* Knee pain, *NKP* Neuropathic-like knee pain, *PRC* Proportional risk contribution

ROC results for the full model including age, gender, body mass index and pain severity

Peripheral = significant injury, nodal osteoarthritis

Central = anxiety, depression, Pain Catastrophising Scale (by highest tertile), fatigue, fibromyalgia

Comorbidities: hyperlipidaemia, diabetes, widespread pain

Pain severity was not included from the second ROC analysis comparing non-NKP with no KP participants

## Discussion

To our knowledge, this is the first community-based study of the prevalence of NKP and associated risk factors. The main findings are as follows: (1) the prevalence of NKP in this sample of people with KP in the Nottingham community is 13.65%; (2) the prevalence of NKP is higher in women and peaks 10 years later than in men; (3) the risk factors associated with definite NKP compared with non-NKP are knee injury; nodal OA; central factors such as depression, anxiety and pain catastrophizing; and comorbidities such as diabetes and hyperlipidaemia; and (4) although contributing factors to NKP are predominantly central, both central and peripheral risk factors contribute equally to non-NKP.

The prevalence of NKP (13.65%) appears higher than in previous questionnaire-based population-wide studies (8.0–8.9%) [25–27]. However, these previous studies defined the prevalence of NKP in the general population, not in a KP population specifically. Conversely, prevalence in our study is also lower than in studies that have reported NKP in symptomatic KOA populations (23%) [28]. This may be because of factors such as different people at risk (KP versus KP plus structural KOA), various definitions of NKP, and sample sizes. In addition to requiring radiographic changes, six of the nine studies in this systematic review used hospital-based samples where more severe cases were included, probably explaining their higher prevalence estimate [29].

Individuals with NKP were more likely to be women and obese (BMI ≥ 30 kg/m^2^) than those without NKP. This accords with previous prevalence studies [29, 30] reporting higher overall neuropathic pain prevalence in women of 8–10.2% compared with 6–7.9% in men [25, 31]. The main risk factors associated with NKP are central factors such as depression, anxiety, pain catastrophising and fatigue. This is supported by a composite measure—the PRC—where contribution from the central risk factors to NKP was greater (8%) than that from peripheral risk factors (3%), whereas the contributions from central (10%) and peripheral (10%) risk factors to non-NKP were equal. It is well known that psychological factors such as depression can influence pain perception and behaviour [32]. It is possible that KP, which contributes to functional limitations, fatigue and possible sleep disturbance, may in turn contribute to lower mood, increased anxiety and worse pain and functional scores, resulting in a complex inter-relationship [33, 34].

This study also showed that fibromyalgia and widespread pain were highly associated with NKP in the community when compared with non-NKP and were still significantly associated, but to a lesser extent, when compared with no-KP participants. Fibromyalgia is often considered a predominantly central ‘top-down’ disorder driven by psychological distress, sleep disturbance and symptoms of anxiety and depression [35], whereas those with neuropathic symptoms have a ‘bottom-up’ disorder driven by peripheral changes and neurogenic damage [36]. The association reported in this study indicates the overlap between these two conditions that share mechanisms of central sensitisation, but causation cannot be established in a cross-sectional study.

Our study also demonstrated that diabetes associates with NKP compared with non-NKP, but not when compared with the no-KP group. Although we did not differentiate type 1 and type 2 diabetes, it is plausible that the metabolic syndrome underlies the onset and progression of neuropathy and obesity, and its consequences are potential driving adverse factors that propagate altered nerve functioning and injury [37].

There are several clinical implications of this project. Whilst KP has often been treated primarily with frontline analgesic agents, the effect sizes for these drugs are small and do not improve over time [38]. This study supports previous findings [39, 40] that there is a neuropathic component to KP that is predominantly driven by central pain sensitisation processes or, more specifically, central risk factors. Considering that the findings that there is a strong contribution and association of central factors such as depression, anxiety and pain catastrophising to definite NKP, these need to be managed correctly in the KP patient population and early stages of the OA process.

There are several limitations to this study. Firstly, the use of self-reported questionnaires might involve recall bias and possible misclassification of self-reported outcomes. Nevertheless, we used a validated questionnaire and involved patient and public volunteers to help optimize clarity and ease of questionnaire use. Secondly, the low response rate (24%) could have resulted in selection bias because those with KP may be more motivated to participate. However, a comparison of KPIC demographic data was made with other local and national UK community cohorts (Appendix), which demonstrated comparability in terms of age, gender distribution and BMI. Thirdly, whilst the questionnaire covered a spectrum of risk factors associated with KP and NKP, we did not measure smoking status, education level or alcohol consumption at baseline. Similarly, we did not have measures of all potential peripheral risk factors (e.g., radiographic OA scores, synovitis and muscle strength). These have minimised the PRC and do not necessarily reflect the real ratio of central and peripheral risk factor contributions. Further study including more comprehensive measurements of both central and peripheral risk factors is therefore warranted. Furthermore, widespread pain was included as a comorbidity rather than as a central factor because it is widely regarded as such in the literature [41, 42]. However, there are limitations in the understanding of pain physiology, and the groups in our analysis (central, peripheral and comorbidity risk factors) may not be entirely mutually exclusive owing to these overlaps, which is a further caveat to our findings.

## Conclusions

In summary, the prevalence of NKP in those with KP in this community sample is approximately 14%. NKP affects more women across all age groups. People with central risk factors such as depression and anxiety and peripheral risk factors such as injury and comorbidities, particularly diabetes, are more likely to have NKP. The results suggest that of the risk factors examined, NKP is predominantly centrally driven, whereas non-NKP is driven equally by both peripheral and central factors. Consideration of NKP characteristics and the balance of central versus peripheral risk factors in individuals with KP could help direct best treatment selection and optimise patient care.

## Appendix

**Table 5:** Comparison of Nottingham Knee Pain and Health in the Community study participants with other population-based cohorts investigation knee pain and knee osteoarthritis in the United Kingdom

Study	Population source, country	Sample size	Age range and mean age (SD)	Female gender, *n* (percentage)	Body mass index, mean (SD)
Knee Pain in the Community (KPIC)^a^	Community, UK	9506	40–90 and 62.10 yr (10.56)	5262 (55.35%)	27.29 (5.29)
Genetics of Osteoarthritis & Lifestyle Study (GOAL)^b^	Community, UK	3710	40–79 and 66.6 yr (7.9)	1780 (48%)	29.3 (5.3)
English Longitudinal Study of Ageing (ELSA)^c^	Community, UK	12,234	50–100 and 65.1 yr (10.2)	6123 (54.5%)	N/A
Health Survey for England (HSfE) 2010/2011^d^	Community, UK	13,983	16–64 and 40 yr	7834 (56%)	Men: 27.4 (0.11) Women: 27.1 (0.11)

^a^ Reference [15]

b Reference [43]

^c^ Reference [44]

^d^ Reference [45]

## Abbreviations

ACR: American College of Rheumatology
aOR: Adjusted odds ratio
BMI: Body mass index
DN4: Douleur Neuropathique 4
GP: General practice
HADS: Hospital and Anxiety Depression Scale
ICOAP: Intermittent and Constant Osteoarthritis Pain measure
KOA: Knee osteoarthritis
KP: Knee pain
mPDQ: Modified painDETECT Questionnaire
NKP: Neuropathic-like knee pain
NREC: Nottingham Research Ethics Committee
OA: Osteoarthritis
PDQ: painDETECT questionnaire
PRC: Proportional risk contribution
QST: Quantitative Sensory Testing
S-LANNS: Self-report version of the Leeds Assessment of Neuropathic Symptoms and Signs
